# Drugs with anti-inflammatory effects to improve outcome of traumatic brain injury: a meta-analysis

**DOI:** 10.1038/s41598-020-73227-5

**Published:** 2020-09-30

**Authors:** Marieke Begemann, Mikela Leon, Harm Jan van der Horn, Joukje van der Naalt, Iris Sommer

**Affiliations:** 1 Department of Biomedical Sciences of Cells and Systems, Section Cognitive Neurosciences, University Medical Center Groningen, University of Groningen, Anton Deusinglaan 2, 9713 AW Groningen, The Netherlands; 2 Department of Neurology, University Medical Center Groningen, University of Groningen, Groningen, The Netherlands

**Keywords:** Immunotherapy, Inflammation, Outcomes research, Clinical pharmacology

## Abstract

Outcome after traumatic brain injury (TBI) varies largely and degree of immune activation is an important determinant factor. This meta-analysis evaluates the efficacy of drugs with anti-inflammatory properties in improving neurological and functional outcome. The systematic search following PRISMA guidelines resulted in 15 randomized placebo-controlled trials (3734 patients), evaluating progesterone, erythropoietin and cyclosporine. The meta-analysis (15 studies) showed that TBI patients receiving a drug with anti-inflammatory effects had a higher chance of a favorable outcome compared to those receiving placebo (RR = 1.15; 95% CI 1.01–1.32, p = 0.041). However, publication bias was indicated together with heterogeneity (I^2^ = 76.59%). Stratified analysis showed that positive effects were mainly observed in patients receiving this treatment within 8 h after injury. Subanalyses by drug type showed efficacy for progesterone (8 studies, RR 1.22; 95% CI 1.01–1.47, p = 0.040), again heterogeneity was high (I^2^ = 62.92%) and publication bias could not be ruled out. The positive effect of progesterone covaried with younger age and was mainly observed when administered intramuscularly and not intravenously. Erythropoietin (4 studies, RR 1.20; p = 0.110; I^2^ = 76.59%) and cyclosporine (3 studies, RR 0.75; p = 0.189, I^2^ = 0%) did not show favorable significant effects. While negative findings for erythropoietin may reflect insufficient power, cyclosporine did not show better outcome at all. Current results do not allow firm conclusions on the efficacy of drugs with anti-inflammatory properties in TBI patients. Included trials showed heterogeneity in methodological and sample parameters. At present, only progesterone showed positive results and early administration via intramuscular administration may be most effective, especially in young people. The anti-inflammatory component of progesterone is relatively weak and other mechanisms than mitigating overall immune response may be more important.

## Introduction

Traumatic brain injury (TBI) remains one of the biggest public health problems and represents a major cause of death or severe disability in young people and adults^1,2^. Benedictus et al.^3^ reported that a significant proportion of patients continues to encounter physical (40%), cognitive (62%), behavioral (55%) and social (49%) problems that may prevent them to return to their former social activity and work after TBI, where severity of injury is associated with higher frequencies^3^. Even patients with good recovery (58%) report problems in one or more of these domains. With a growing awareness of the deleterious effects of TBI, early interventions to ameliorate the effects and improve outcomes are at the heart of science^4^.

In the central nervous system (CNS), an inflammatory response occurs directly after TBI, involving both resident and peripheral immune cells as measured both in blood and in the brain^5,6^. The effects of this accompanying inflammatory reaction are considered to have both beneficial and harmful aspects. Immune activation is needed for neuro-reparative mechanisms after brain injury, defends against invading pathogens and repairing damaged brain tissue. However, ongoing neuro-inflammatory responses also contribute to the development of cerebral edema and swelling, a breakdown of the blood–brain barrier, and delayed neuronal cell death^7–9^. Inflammatory mediators (bioactive lipids, cytokines, and chemokines) have been investigated for their protective and deleterious roles after TBI. So far, it is not fully understood which inflammatory mediators need to be targeted to obtain the optimal therapeutic effect^1,9^.

The observed association between TBI and neuroinflammation has stimulated research on the application of immune modulating components as a potential treatment option. Animal studies testing the efficacy of agents with anti-inflammatory actions have provided promising leads for clinical trials^6^. For example, administration of erythropoietin, which has mild anti-inflammatory properties but also many other actions, significantly reduced edema and improved cognitive function in a mouse experimental TBI model^10^. With the same drug, reduction of hippocampal neuron loss and enhanced neurogenesis in the injured cortex and hippocampus has been observed, which led to improved sensorimotor function and spatial learning in mice^11,12^. Other components with anti-inflammatory properties have shown similar beneficial effects, yet results vary according to the investigated injury model and/or treatment window^6^. Moreover, translating positive findings from animal studies to clinical results has proven difficult, with some studies finding positive results for immune mediating interventions in patients^13,14^, while other results are negative^15^.

A structured overview of the effects of drugs with anti-inflammatory properties after TBI may help to determine possible future clinical studies and eventually improve care for TBI patients. Current study aims to provide a quantitative summary of randomized controlled studies in patients with TBI providing drugs with anti-inflammatory aspects. Many pharmacological agents have at least some degree of anti-inflammatory action, either as primary function, or as for others it is only one of their mechanisms of action. In order to be as inclusive as possible, we selected all agents that have some anti-inflammatory aspects, as demonstrated in (rodent) experimental settings. This results in a very broad range of agents, which have one important feature in common: their anti-inflammatory effects.

## Method

### Search strategy

A systematic literature search was performed using PubMed, Cochrane Library and the National Institutes of Health Website ClinicalTrials.gov, following PRISMA guidelines. To identify other potentially eligible studies, we searched through citations and references from all relevant publications for cross-references. Search cut-off date was August 2019. There were no year or language restrictions. Following a previous study^16^ investigating the effects of anti-inflammatory agents in schizophrenia treatment, the search strategy consisted of the combination of the following keywords: traumatic brain injury OR head injury OR head trauma AND the specific pharmacological components: NSAIDs (non-steroidal anti-inflammatory drugs, i.e. aspirin, celecoxib, ibuprofen, diclofenac, naproxen), devunetide, EPA (eicosapentaenoic acid) and DHA (docosahexaenoic acid) fatty acids, estrogen, minocycline, NAC (*N*-acetyl-l-cysteine) and corticosteroids (i.e. prednisone, prednisolone, hydrocortisone, methylprednisolone, dexamethasone, cortisone, triamcinolone, betamethasone), transplantation adjuncts (tacrolimus, cyclosporine, everolimus, serolimus, mycophenolate mofetil), cytostatics (bexarotene, methotrexate, cyclophosphamide), melatonin, progesterone, and erythropoietin^16^. Progesterone and erythropoietin were added as search terms after a manual search of citations from retrieved studies, as anti-inflammatory mechanisms have been reported for both components^10,17,18^.

### Inclusion criteria

Studies and trials meeting the following criteria were included.

1. Study design: randomized, single or double-blinded, placebo-controlled trials.
2. Condition: patients with a clinical diagnosis of TBI or diffuse axonal injury (DAI) regardless of its severity (mild, moderate, severe).
3. Intervention: pharmacological components with confirmed anti-inflammatory mechanisms and effects. Agents could be administered in any dose, by any route, for any duration of time initiated within 24 h after TBI.
4. Study outcome: neurologic and functional outcome measured by the Glasgow Outcome Scale (GOS) or Glasgow Outcome Scale-Extended (GOS-E).
5. Studies reported sufficient information to compute common effect sizes (ES) statistics, or authors were able to provide data upon request.

### Outcome measures

The main outcome was functional outcome as measured using the Glasgow Outcome Scale (GOS) or Extended Glasgow Outcome Scale (GOS-E), which scores are dichotomized to favorable or unfavorable outcome. The GOS rates patient status into one of the five ordinal outcome categories: dead, vegetative state, severe disability, moderate disability or good recovery. Because of the ceiling effects, the GOS has been modified into GOS-Extended to address these shortcomings^19^. The GOS-E provides eight categories by splitting severe disability, moderate disability, and good recovery into a lower and upper category. The dichotomization into favorable (good recovery and moderate disability) and unfavorable (severe disability, vegetative state, and death) category has been used extensively in RCTs. Effects are usually estimated by means of the proportion of favorable outcome in each group.

### Study selection and data extraction

Titles and abstracts were screened for studies that did fulfilled the inclusion criteria. Studies that appeared relevant after screening were selected for further full-text review. The following variables were extracted from each study: author, year of publication, study design, condition, and drug intervention, patients' characteristics, for treated and control group separately: number of patients enrolled and percentage of males in each group, age [as mean (M) and standard deviation (SD) or median (Me) and interquartile range (IR)]. In case N of patients enrolled for the treatment and control group was unclear, we used N of patients included in the final analysis. Additionally, Glasgow Coma Scale scores at initial assessment and treatment protocols (dosage, the way of application, and treatment duration) were extracted.

Studies differed in terms of the number of follow-up measurements. In line with previous literature, we preferably used the outcome reported at 6 months after the intervention or end of the follow up period^20^. Furthermore, Wright et al.^21^ reported outcomes separately for a moderate and a severe TBI group. As the moderate group was small in sample sizes, outcomes were combined in our analyses. The same was done for Robertson et al.^22^, who compared two different dosage regimens of erythropoietin (Epo) to one placebo group.

### Statistical analysis

Effect sizes were computed using Comprehensive Meta-Analysis Version 2.0, Biostat^23^. Analysis was run on all studies eligible for quantitative analysis. Dichotomous outcomes comparing recovery rates between treatment and control groups were analyzed as the risk ratio (RR), testing the null hypothesis that there are no differences (true RR is 1.0), while a RR greater than 1 indicates that patients in the intervention group were more likely to improve than those treated with placebo.

All included studies were combined to calculate a mean weighted ES for the effect on functional outcome, using a random effects model. Individual components were also separately evaluated. To investigate whether studies could be combined to share a common population effect size, the Q-value and I^2^-statistic were evaluated for each analysis. The Q-statistic tests the existence of heterogeneity and displays a chi-square distribution with k − 1 degrees of freedom (k = number of study samples). Q-values higher than the degrees of freedom (df) indicate significant between-studies variability. I^2^ indicates the proportion of the observed variance in true effect sizes rather than sampling error, ranging from 0 to 100%. Heterogeneity can be interpreted as low (≤ 25%), moderate (25–50%) or high (> 50%). Further investigating potential heterogeneity between studies, moderator analyses were performed to evaluate whether study characteristics covaried with intervention effectiveness; including time to treatment after injury (hours), intervention duration (hours) and age (years) and gender distribution (% male) of the included study samples. Additionally, subgroup analyses were performed to evaluate whether the effect on favorable outcome differed between the pharmacological agents. When relevant and in case of sufficient study samples, moderator analyses were repeated per subgroup, in addition to stratifying subgroup analyses by administration route of the individual drug components to examine potential differences in the effect on favorable outcome. *p* values < 0.05 were considered significant.

When interpreting meta-analytic outcomes, the possibility of an upward bias of the calculated effect sizes due to the omission of unpublished, nonsignificant studies must be taken into account. Potential publication bias was investigated by means of a visual inspection of the funnel plot and Egger’s test^12^ was evaluated when appropriate (i.e., analysis included a range of study sizes, with at least one of ‘medium’ size (p < 0.05 two-tailed). Moreover, the fail-safe number of studies (NR) was calculated, providing an estimate of how many unpublished null findings would be needed to reduce an observed overall significant result to non-significance (the fail-safe number should be 5k + 10 or higher [k = number of studies in a meta-analysis] to rule out a file drawer problem.

## Results

### Search results

**Figure 1:**
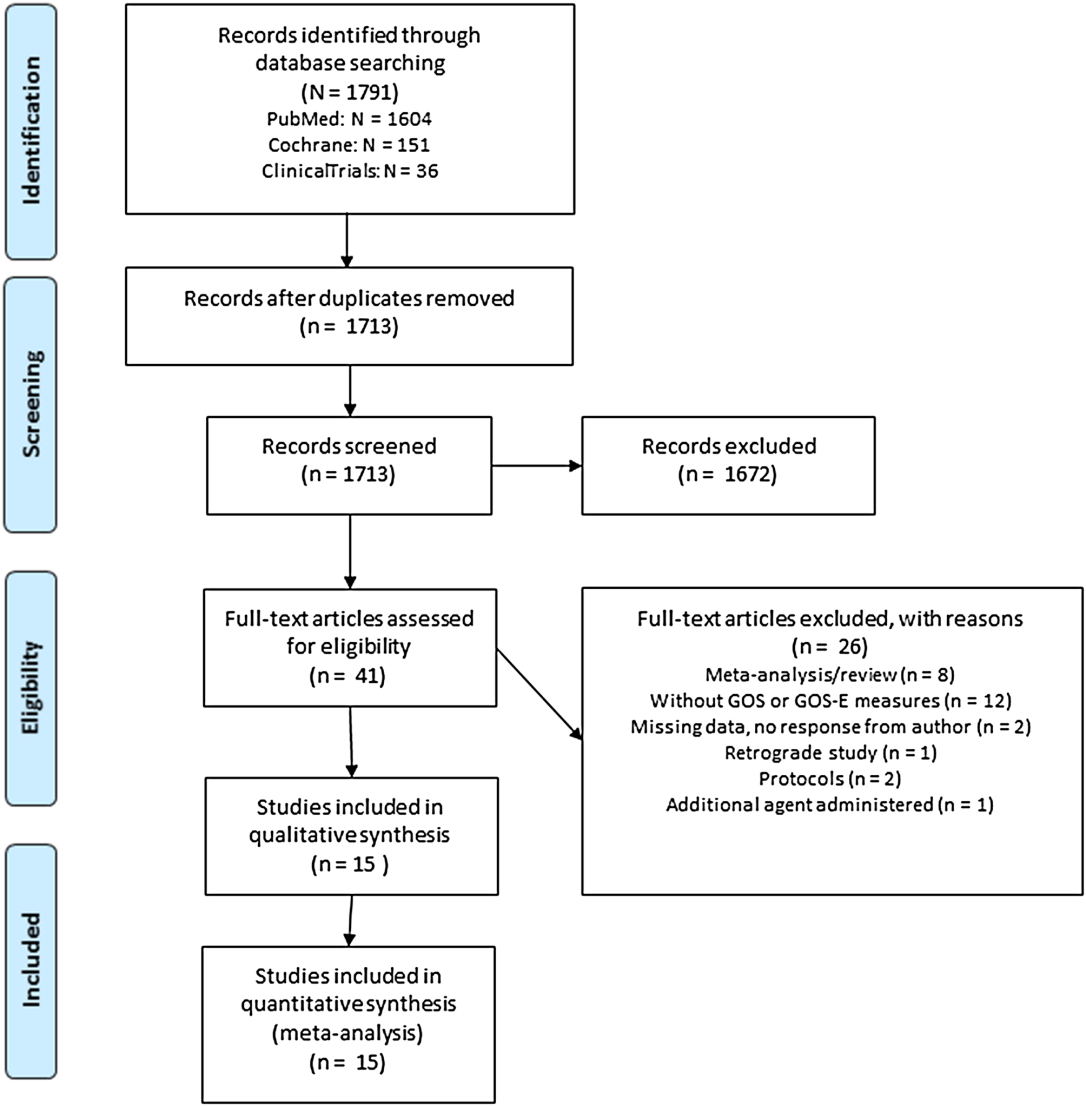
Flow diagram of the systematic search and study selection.

The search strategy yielded 1791 results (see Fig. 1). After screening and full-text reading, studies meeting defined criteria were included for quantitative synthesis. In total 15 studies (cyclosporine: three studies; erythropoietin four studies; progesterone eight studies), including 3734 patients were finally eligible for quantitative analysis. Corticosteroids have been previously evaluated in a Cochrane review^24^ (updated in 2009), including the large CRASH study that dominated the results^24–26^. Moreover, as we did not identify any new corticosteroid studies, it was decided not to pool these results in current meta-analysis. The characteristics of the fifteen trials included in this meta-analysis are presented in Table 1 (see Appendix Table E1 for the number of patients with favorable outcome within each individual study, separately for treatment and control group).

**Table 1:** Overview of the included studies.

Study (author, year)	Condition		Study design	No. of patients enrolled (male %)		Mean age (SD or IQR)		GCS included patients	Intervention	Outcome included in meta-analysis
				Treatment	Control	Treatment	Control			
**Progesterone**										
Wright^21^	TBI	RCT DB		77 (70%)	23 (71%)	35.3 (14.3)	37.4 (17.4)	4 < GCS < 12	Loading dose 0.71 mg/kg at 14 mL/h first hour, then reduced to 10 mL/h to deliver 0.5 mg/kg per hour for next 11 h. Five 12-h maintenance infusions delivered at 10 mL/h for total of 3 days of treatment	GOS-E 30 days
Xiao^27^	TBI	RTC DB		82 (70%)	77 (74%)	30 (11)	31 (9)	GCS ≤ 8	1 mg/kg via intramuscular injection, once per 12 h for 5 consecutive days	GOS 6 months
Aminmansour^28^	TBI/DAI	Simple random sampling		20 (80%)	20 (60%)	28.0 (7.4)	31.5 (8.2)	GCS ≤ 8	Within 8 h after injury, 1 mg/kg of progesterone intramuscularly every 12 h for 5 days	GOS 3 months
Shakeri^29^	DAI	RCT SB		38 (100%)	38 (100%)	34.0 (12.5)	34.7 (12.9)	GCS ≤ 8	1 mg/kg every 12 h for 5 days	GOS 3 months
Skolnick^30^	TBI	RCT DB		591 (79%)	588 (79%)	35 (23–51)	34 (24–50)	GCS ≤ 8	Loading dose 0.7 mg/kg per 1 h intravenously, followed by 0.50 mg/kg per 1 h for 119 h	GOS 6 months
Wright^31^	TBI	RCT DB		422 (73%)	440 (74%)	36 (17–93)	36 (17–94)	4 < GCS < 12	Loading dose within 4 h of injury 14 ml/h for 1 h, then 10 ml/h for 71 h, dose then tapered by 2.5 ml/h every 8 h for total treatment of 96 h	GOS-E 6 months
Sinha^37^	TBI	RCT (blinding nr)		26 (76%)	27 (85%)	33.7 (10.9)	33.9 (11.2)	4 < GCS < 8	Within 8 h of injury, 1.0 mg/kg via intramuscular injection, once every 12 h for 5 consecutive days	GOS 6 months
Soltani^32^	DAI	RCT SB		24 (100%)	24 (100%)	27.9 (1.4)	30.4 (2.5)	3 < GCS < 12	1 mg/kg every 12 h for 5 days	GOS-E 6 months
**Erythropoietin**										
Robertson^a^^22^	TBI	RCT with factorial design		Epo1 = 35 (90%) Epo2 = 57 (86%)	89 (86%)	Epo1 = 32 (23–48) Epo2 = 29 (23–47)	30 (22–44)	GCS > 3	Epo1: one dose of 500 IU/kg within 6 h of injury, then two additional doses every 24 h (changed in 2009 because of safety) Epo2: one dose of 500 IU/kg, within 6 h of injury	GOS 6 months
Nichol^33^	TBI	RCT DB		308 (84%)	298 (83%)	30.5 (22.4–47.5)	30.5 (22.9–48.3)	3 < GCS < 12	Within 24 h of injury, 40,000 IU subcutaneously, then weekly for max of 3 doses	GOS-E 6 months
Li^34^	TBI	RCT DB		79 (65%)	80 (75%)	43.3 (10.1)	41.1 (9.4)	GCS ≤ 8	Subcutaneous injection of Epo (100 units/kg) in 5 doses (day 1, 3, 6, 9, 12)	GOS 3 months
Bai and Gao^35^	TBI	RCT DB		60 (68%)	60 (73%)	44.5 (11.4)	43.1 (10.9)	GCS < 8	Subcutaneous injection of RHE (6000 IU) within 2 h of admission, then same dosage on day 3, 5, 10 and 15	GOS 10 weeks
**Cyclosporine**										
Hatton^36^	TBI	RCT DB		32 (75%)	8 (100%)	I = 29 (6.0) II = 32 (14.6) III = 23 (8.2) IV = 34 (14.8)	6 (6.6)	4 < GSC < 8	4 cohorts, different dosing (3 cohorts with 0.625–2.5 mg/kg/dose every 12 h for 72 h (6 doses each)) 1 cohort with 2.5 mg/kg loading dose, then 5 mg/kg/day continuous infusion for 72 h	GOS 6 months
Mazzeo^15^	TBI	RCT Double-blind		37	13	34 (16)	29 (14)	3 < GSC < 8	Within 12 h of injury 5 mg/kg of CsA, over 24 h	GOS 6 months
				(82% males in total sample)						
Aminmansour^59^	DAI	RCT DB		50 (90%)	50 (86%)	29.9 (8.7)	31.3 (10.7)	GCS ≤ 10	Within 8 h of injury 5 mg/kg, via 250 ml dextrose water for 24 h	GOS-E 6 months

*SD* standard deviation, *IQR* inter quartile range, *TBI* traumatic brain injury, *DAI* diffuse axonal injury, *RCT* randomized controlled trial, *DB* double-blind, *SB* single-blind, *GCS* Glasgow Coma Scale, *GOS* Glasgow Outcome Scale, *GOS-E* Extended Glasgow Outcome Scale, *IU* international unit, *RHE* recombinent human erythropoietin, *n.r.* not retrieved from the article.

^a^Not reported for final sample, numbers are retrieved from demographics Table describing the total number of enrolled patients.

### Overall meta-analysis

**Figure 2:**
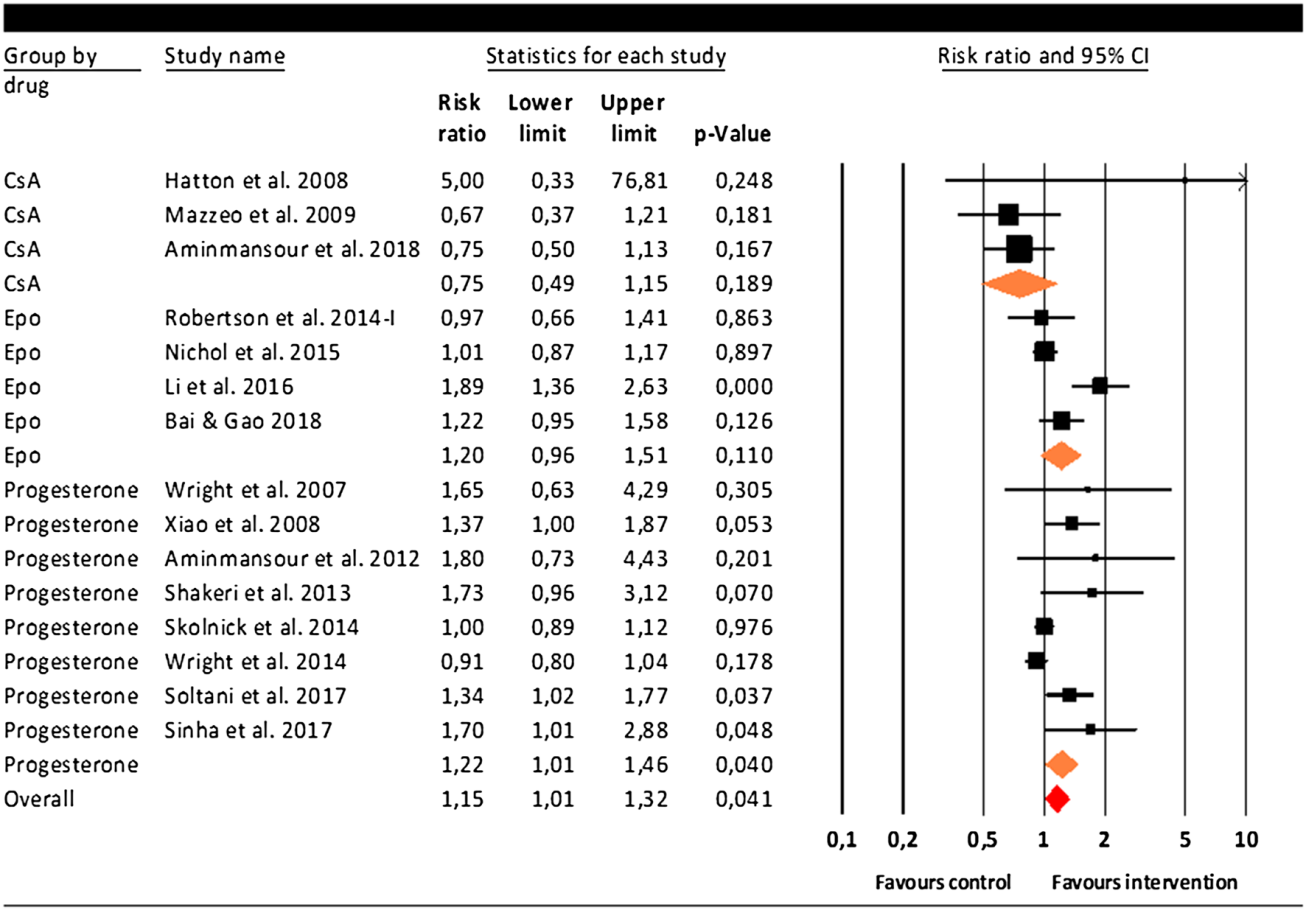
Meta-analysis including 15 studies evaluating the effect of drugs with anti-inflammatory properties on a favorable outcome in TBI patients.

The overall analysis, including 15 studies, showed that the mean risk ratio was 1.15 (95% CI 1.01–1.32, p = 0.041), indicating that drugs with anti-inflammatory properties as received in the intervention group increased the chance of a favorable outcome relative to placebo. As shown in Fig. 2, three studies showed a significant individual RR (p < 0.05) favoring the treatment group^32,34,37^.

**Figure 3:**
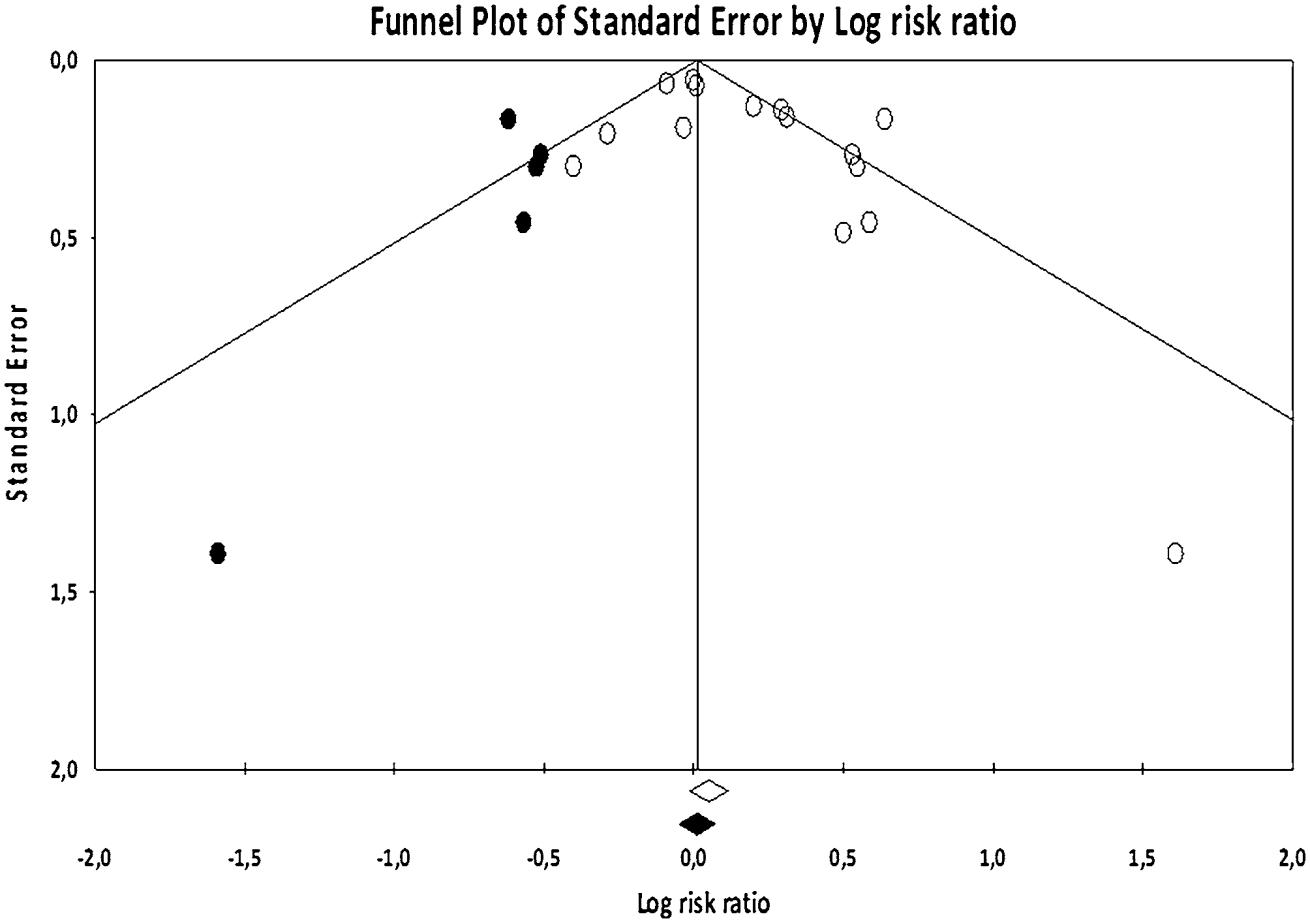
Funnel plot showing publication bias in the overall analyses (15 studies), including imputed studies (black circles).

The Q-value of 39.24 (p < 0.001) indicated that the variability among studies was higher than would be expected due to sampling error alone and that there is evidence of variance in true effects. Heterogeneity was high (I^2^ = 64.32%), indicating that over 64% of the dispersion reflects the differences in true effect sizes, while the remaining 36% can be attributed to random sampling error. Moreover, publication bias was indicated by the significant Egger’s test (t = 2.23, p = 0.043) and when observing the funnel plot, the trim-and-fill procedure suggested four studies were potentially missing, reducing the RR to 1.02 (95% CI 0.96–1.09, see Fig. 3). The number of unpublished negative studies needed to render our significant RR of 1.15 nonsignificant was N_R_ = 26.

When further investigating heterogeneity, moderator analyses showed that the treatment effect of drugs with anti-inflammatory properties on favorable outcome was not associated with intervention duration, age or gender distribution (% male). Time to treatment after injury was evaluated using a categorical approach, as 10 out of 15 studies reported a similar administration time (8 h after injury). The effect of drugs with anti-inflammatory properties on favorable outcome did not differ between the 11 trials with a faster administration time (within 8 h of injury) and the four trials with a longer administration time (Q(1) = 0.665, p = 0.415). However, while the combined RR of 1.05 for studies with a longer administration time did not reach significance [subtotal n = 876; 95% CI 0.80–1.38, p = 0.153; Q(3) = 4.73, p = 0.193; I^2^ = 36.60%], the trials with a faster administration resulted in a combined RR of 1.20 favoring the effect of drugs with anti-inflammatory properties (subtotal n = 2858; 95% CI 1.02–1.42, p = 0.033) but with significant heterogeneity (Q(10) = 34.49, p < 0.001; I^2^ = 71.01%).

### Subgroup analyses

**Figure 4:**
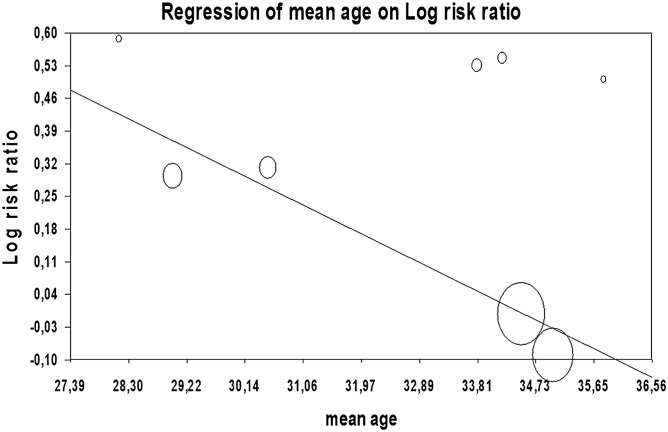
Moderator analysis for the 8 progesterone studies, risk ratio covaries with mean age of the included study samples.

The effect on favorable outcome did not differ across the three drugs with anti-inflammatory agents properties (Q(2) = 4.54, p = 0.103). However, when evaluating RR according to subgroup, progesterone showed a significant risk ratio of 1.22 (95% CI 1.01–1.47, p = 0.040), see Fig. 2. Notably, the Q-statistic was significant and heterogeneity was high (Q(7) = 18.88, p = 0.009; I^2^ = 62.92%). Publication bias was indicated by the significant Egger’s test (t = 4.45, p = 0.004) and when observing the funnel plot, the trim-and-fill procedure suggested four studies were potentially missing, reducing the RR to 1.10 (95% CI 0.93–1.28). Fail-safe N_R_ was 12. Moderator analyses indicated that age was significantly related to the effect of progesterone on favorable outcome, with lower RR in study samples with a higher mean age (Q = 9.92, slope − 0.07, p = 0.002; see Fig. 4). Gender distribution (%) did not moderate this effect (Q = 1.64; p = 0.200). Moderator analyses could not be performed for time to treatment nor treatment duration as included studies showed little variation in those parameters. The positive effect of progesterone did significantly differ in terms of administration route (Q = 15.73, p ≤ 0.001). Although based on a relatively small number of studies, results were homogeneous and showed that progesterone only showed a beneficial effect on outcome when administered intramuscularly (RR 1.41, 95% CI 1.41–1.17, p < 0.001; I^2^ = 0%) but not when patients received progesterone intravenously (RR 0.97, 95% CI 0.97–1.05, p = 0.420; I^2^ = 9.72%).

For erythropoietin (four studies), the RR was similar to progesterone yet did not reach significance (RR 1.20; 95% CI 0.96–1.52; p = 0.110; Fig. 2). There was a significant level of heterogeneity across the erythropoietin studies (Q(3) = 12.81, p = 0.005; I^2^ = 76.59%). Cyclosporine (three studies) did not show a significant effect with low heterogeneity (RR 0.75; 95% CI 0.49–1.17, p = 0.189; Q(2) = 2.00. p = 0.369; I^2^ = 0%; Fig. 2). Due to the low number of studies included for both erythropoietin and cyclosporine, no moderator analyses could be performed.

## Discussion

This study investigated if agents with anti-inflammatory properties can help to ameliorate functional and neurological recovery in patients suffering from consequences of traumatic brain injury (TBI). In total, 15 studies were summarized for quantitative analysis to gain perspective on how current literature provides evidence for efficacy of anti-inflammatory drugs, including three different components: progesterone, erythropoietin and cyclosporine. The overall effect of these drugs with anti-inflammatory properties turned out to be significant yet small (RR = 1.15), with considerable heterogeneity among studies. A faster time to treatment after injury could be a factor of interest for the beneficial effects of agents with anti-inflammatory properties in TBI patients, although this did not reduce heterogeneity. When drug type was tested for efficacy in improving outcome, progesterone showed a small effect size, yet heterogeneity among studies remained high. As a small improvement is easily out-weighed by the risk of side-effects, which was not tested in this study, positive recommendation for clinical use is premature. Furthermore, publication bias was observed in the overall analysis and the studies reporting progesterone efficiency, which reflects proneness to publication of studies only reporting positive results. No effects were found for erythropoietin and cyclosporine.

This is the first quantitative analysis that provides evidence on the overall efficacy of drugs with anti-inflammatory properties in TBI. In order to be as inclusive as possible, we broadly selected agents that have shown some anti-inflammatory effects, which resulted in a total number of 15 clinical trials on three different components. The overall positive RR of 1.15 was small yet significant, but results need to be interpreted with caution due to the publication bias that was indicated as well as the high heterogeneity among the included studies. Moreover, progesterone was the most investigated component and two of those trials had an exceptionally large sample size (n > 850). While intervention duration, age, or gender distribution did not moderate the overall effect of drugs with anti-inflammatory properties on a favorable outcome in TBI, we did find that efficacy may be dependent on time of treatment. Studies administrating an agent with anti-inflammatory effects within 8 h after injury showed a significant combined RR of 1.20 as compared to placebo, while trials with a longer administration time resulted in a lower and insignificant combined RR of 1.05. However, this latter result was based on a relatively small number of four trials (subtotal n = 876 participants) and we must note that heterogeneity remained high among the faster administration trials. Although issues with the quality and clinical relevance of the preclinical animal models have been noted, animal studies have also suggested that a faster administration time can positively moderate treatment outcome^38^. For example, the strongest anti-inflammatory effect of progesterone can be expected when dosed 1 h after the injury^39,40^, or within 6 h for erythropoietin^41^.

### Progesterone

Progesterone has multiple anti-inflammatory actions and its effects on brain edema involves complex mechanisms and inflammatory mediators^18,42–44^. Multiple animal studies investigating the efficacy of progesterone on improving outcome after TBI have yielded positive results^40,42,43^. Combining eight studies in patients with TBI, our results show that progesterone treatment can elicit a small positive effect on favorable outcome, which is in line with a previous meta-analysis^45^. We observed high heterogeneity between the included studies and the analysis stratified by administration route indicated that the effect of progesterone was mainly observed when administered intramuscularly (RR 1.41); not when patients received progesterone intravenously (RR 0.97)^45^. Notably, intramuscular administration was mainly implemented by the relatively smaller studies, while the studies with larger sample sizes investigated the intravenous route. Moreover, we found that age covaried with the effect of progesterone on favorable outcome, with lower RR in younger TBI patient samples. Progesterone has previously shown efficacy in studies on middle-aged and aged rats^40,46^. Unfortunately, moderator analyses could not be performed for time to treatment administration and treatment duration, as the included studies showed little variation in those parameters. For example, all trials implemented treatment 8 h after injury accept for Wright et al. who applied progesterone after 11 h^21^. Further research is necessary to evaluate whether a faster administration time could potentially improve the effect of progesterone on treatment outcome. Appropriate clinical trial design needs careful consideration to confirm intramuscular progesterone as preferred route and the potential moderating role of age and, also elaborating on previous negative progesterone, further investigate factors such as optimal treatment duration and dosage trials^21,30^.

### Erythropoietin

Animal studies have reported on the efficacy of erythropoietin against multiple early mediators of secondary brain injury, including anti-inflammatory effects in terms of reducing proinflammatory cytokines as well as increasing anti-inflammatory cytokines in brain tissue^10^. In turn, these anti-inflammatory effects proposedly contribute to the erythropoietin-induced neuroprotective effects after TBI seen in preclinical studies^10,47^. Contrasting animal work, the present study (including four RCTs) did not confirm the effectiveness of erythropoietin in improving neurological recovery. The RR for erythropoietin was similar to progesterone yet did not reach significance, which could indicate a power problem. This negative finding corroborates a previous meta-analysis by Lee et al. including three RCTs^48^, who did show that the use of EPO may prevent death following TBI. The study by Li et al. was the only positive erythropoietin study in our meta-analysis, with a large effect size of 1.89^34^. Compared to the other three trials with null-results, Li et al. administered the first dose relatively fast, within 2 h of admission^34^. This treatment timeframe was similar to the recent study by Bai and Gao, who sampled a slightly lower number of participants and found a positive yet lower effect size of 1.22 that did not reach significance (*p* = 0.126)^35^. Animal studies also suggest that earlier administration, preferably within 6 h, seems to elicit the most effect, although positive effects have also been shown when administered one-day post injury^11,41^. Although we could not perform a moderator analysis for erythropoietin dosage within our meta-analysis, Gantner et al. ran post hoc analyses for the large EPO-TBI trial by Nichol et al. and reported that the strongest benefit was seen in patients who received two doses of erythropoietin^33,49^.

### Cyclosporine

In spite of existing evidence for cyclosporine anti-inflammatory action, only few clinical trials with this component have been performed to test its effect on neurological recovery^50,51^. So far, its neuroprotective role has mainly been tested in animal models, which underline the importance of initiating administration of cyclosporine as soon as possible following TBI^52^. We could only include three studies and found that the overall effect size did not favor cyclosporine treatment. Although none of the individual trials reached significance, two studies showed a RR below 1.00 while Hatton et al.^36^ was an exceptionally large positive outlier (RR 5.00), potentially because none of the patients gained a favorable outcome in the much smaller control group (n = 8, versus a treatment group of n = 32). As cyclosporine is listed as a potential treatment therapy for TBI, our negative results urge caution and highlight the need for additional studies on this component^53^.

### Limitations

The pathogenesis of TBI is complex and outcome is determined by different mechanisms and level of severity, neuroanatomical location and distribution of brain lesions. Each TBI patient has an unique pattern of cerebral damage. This heterogeneity of injury patterns make it even harder to develop effective therapies that could target multiple cellular and molecular events and will be applicable for each patient suffering from TBI^1^. The diversity and complexity could be the reason for several failed clinical trials despite successful procedures in experimental settings^2^. Therefore, only few trials could be included per component investigated, which limits the impact of our findings. Regarding the chosen outcome measure, the Glasgow Outcome Scale (GOS) and Extended Glasgow Outcome Scale (GOS-E), are confirmed to be reliable and most accepted instrument available^19^. In addition, researchers have found a significant correlation between performance on the neuropsychological measures and the GOS-E scores^54,55^. However, was recommended by the TBI Clinical Trials Network Outcome Measure Subcommittee^54^, a single, global measure is less suitable in evaluating multiple domains of recovery from TBI. Within our meta-analysis, only two out of 15 studies had included additional neuropsychological testing. In future studies, more sensitive outcome measures should be implemented that can differentiate degrees of functional outcome.

Modulation of the inflammatory pathway may also be an important mechanism underlying the effects of other agents that have previously shown therapeutic benefit in TBI treatment, as described in an extensive review by Bergold^56^. For example statin use, with a double‐blind RCT in 36 patients with moderate and severe TBI showing that rosuvastatin reduces disability scores together with TNF‐α, no effect was found on IL‐1ß, IL‐6, and IL‐10^57^. Moreover, modification of the neuroinflammatory response has also been found for recombinant human IL1ra (rhIL1ra, anakinra), in a phase II, open label, randomized-control study including 20 patients with severe TBI^58^. More knowledge on the anti-inflammatory mechanisms behind such pharmacological components can help to improve (pre)clinical testing of agents in the search for effective TBI treatment.

## Conclusion

Based on the available literature, it is too early to draw any conclusions on the efficacy of drugs with anti-inflammatory properties in the treatment of TBI patients. Progesterone showed some promising results in improving the outcome of TBI patients yet verification is needed. To strengthen the body of research on TBI treatment, future well-designed trials are needed to evaluate the factors that could potentially moderate treatment effects, including the initial time of treatment after injury, administration route but also patient characteristics such as age of the patient suffering from TBI^59^.

## Supplementary information

Supplementary Table E1.
