# Supplementary material for: Drugs with anti-inflammatory effects to improve outcome of traumatic brain injury: a meta-analysis
Source: Sci Rep. 2020 Sep 30;10:16179. doi: 10.1038/s41598-020-73227-5 (PMC7528105; doi:10.1038/s41598-020-73227-5)
Supplement: Supplementary file 1 — Supplementary Table E1. [file 41598_2020_73227_MOESM1_ESM.docx]

**Drugs with anti-inflammatory effects to improve outcome of traumatic brain injury:**

**a meta-analysis**

# Marieke Begemann^1^, Mikela Leon^1^, Harm Jan van der Horn^2^,

# Joukje van der Naalt^2^, Iris Sommer^1^

Online Supplementary Information

# Appendix: Table E1. *N of patients with favorable outcome, separately for treatment and control group.*

| **Study** | **Favorable outcome** | **Total** | **Favorable outcome** | **Total** |
| --- | --- | --- | --- | --- |
|  | **Cyclosporine** | | **Control** | |
| Mazzeo, 2009 | 14 | 34 | 8 | 13 |
| Hatton, 2008 | 7 | 20 | 0 | 6 |
| Aminmansour, 2018 | 21 | 50 | 28 | 50 |
| **Subtotal** | 42 | 104 | 36 | 69 |
|  | **Erytropoietin** | | **Control** | |
| Robertson, 2014a | 17 | 35 | 17 | 44 |
| Robertson, 2014b | 17 | 57 | 17 | 45 |
| Li, 2016 | 54 | 75 | 27 | 71 |
| Nichol,2015 | 168 | 302 | 162 | 294 |
| Bai & Gao, 2018 | 44 | 60 | 36 | 60 |
| **Subtotal** | 300 | 529 | 259 | 514 |
|  | **Progesterone** | | **Control** | |
| Shakeri,2013 | 19 | 38 | 11 | 38 |
| Aminmansour, 2012 | 9 | 20 | 5 | 20 |
| Xiao, 2008 | 48 | 82 | 33 | 77 |
| Skolnick, 2014 | 298 | 591 | 297 | 588 |
| Wright, 2014 | 213 | 442 | 232 | 440 |
| Soltani, 2017 | 19 | 20 | 17 | 24 |
| Wright, 2007 | 21 | 70 | 4 | 22 |
| Sinha, 2017 | 17 | 23 | 10 | 23 |
| **Subtotal** | 644 | 1286 | 609 | 1232 |
| **Total** | **986** | **1919** | **904** | **1815** |
